# The Neuropeptides of Ocular Immune Privilege, α-MSH and NPY, Suppress Phagosome Maturation in Macrophages

**DOI:** 10.4049/immunohorizons.1800049

**Published:** 2018-11

**Authors:** Isaac J. Benque, Pu Xia, Robert Shannon, Tat Fong Ng, Andrew W. Taylor

**Affiliations:** Department of Ophthalmology, Boston University School of Medicine, Boston, MA 02118

## Abstract

The ocular microenvironment has evolutionarily adapted several mechanisms of immunosuppression to minimize the induction of inflammation. Neuropeptides produced by the retinal pigment epithelial cells regulate macrophage activity. Two neuropeptides, α-melanocyte–stimulating hormone (α -MSH) and neuropeptide Y (NPY), are constitutively expressed by the retinal pigment epithelial cells. Together these two neuropeptides induce anti-inflammatory cytokine production in endotoxin-stimulated macrophages and suppress phagocytosis of unopsonized bioparticles. These neuropeptides do not suppress the phagocytosis of opsonized bioparticles; however, they do suppress phagolysosome activation or formation. In this report, we studied the possibility that α-MSH with NPY suppress phagosome maturation within macrophages using opsonized OVA-coated magnetic beads to isolate and analyze the phagosomes. The magnetic bead–containing intercellular vesicles were isolated and assayed for Rab5, Rab7, LAMP1, Ia^d^, and OVA. The macrophages cotreated with α-MSH and NPY were suppressed in Rab7 recruitment to the phagosome with suppression in LAMP1 expression but not in Ia^d^ expression. The results demonstrated that the α-MSH/NPY cotreatment suppressed phagosome maturation. In addition, the a-MSH/NPY–cotreated macrophages were suppressed in their ability to Ag stimulate CD4^+^ T cell proliferation. These results imply a potential mechanism of ocular immune privilege to divert Ag processing to prevent autoreactive effector T cells from binding their target cognate Ag within the ocular microenvironment.

## INTRODUCTION

The regulation of immune responses by neuropeptides within the ocular microenvironment holds an important role in the mechanisms of immune privilege. Within the healthy eye, neuropeptides help prevent activation of macrophages and microglial cells to mediate inflammation (1). The retinal pig-ment epithelial cells (RPE), a monolayer of cells that forms the posterior blood barrier upon which the photoreceptors rest, produce neuropeptides that modulate the activity of macrophages (2, 3). Two of these neuropeptides are α-melanocyte–stimulating hormone (α-MSH) and neuropeptide Y (NPY) (3). Through these two neuropeptides, RPE suppress proinflammatory activity while promoting regulatory activity in stimulated macrophages (3–6). The macrophages have an unusual pattern of coexpressing NO synthase2 and arginase-1 while producing IL-10 and TGF-b (3). In addition, microglial cells in the healthy neuroretina have similar alternatively activated myeloid cell characteristics (3). In eyes with wounded RPE, the RPE do not mediate the alternative activation but promote macrophage/microglial cell differentiation into proinflammatory M1 or wound repairing/anti-inflammatory M2 cells (3, 5). The wounded RPE monolayer continues to produce NPY and is greatly diminished in α-MSH production (3). These finding have demonstrated the importance of specific neuropeptides in the mechanisms of RPE-mediated immunosuppression. Moreover, it suggested that the coexpression of α-MSH and NPY is a way of modulating macrophage activity to suppress and prevent inflammation and to promote immune regulation and tolerance.

Individually these neuropeptides have differing and sometimes contradictory effects on immune cells and on immune activity. The melanocortin receptors for α-MSH are Gs coupled, with link-age to selective JAK/STAT and ERK1/ERK2 intracellular signaling pathways (7–11). The effects of α-MSH on monocytes and macrophages are well characterized as anti-inflammatory. This neuropeptide not only suppresses the production of proinflam-matory cytokines but blocks the activation of transcription factors required for inflammatory cytokine gene activation (12–15). In addition, it promotes the production of anti-inflammatory cyto-kines IL-10 and TGF-β1 along with promoting its own production and receptor expression (2, 16–18). In contrast, the effects of NPY on monocytes and macrophages are not as clear as the effects of α-MSH. Monocyte adherence, chemotaxis, and IL-1β production are enhanced by NPY; however, NPY can induce the production of anti-inflammatory TGF-b1 (19–21). The effects of NPY on phagocytosis is equally contradictory. Depending on what the macrophage is phagocytizing, inert latex beads or specific pathogens, NPY will either enhance or suppress phagocy-tosis (22–26). In addition, unlike a-MSH, NPY enhances the generation of reactive oxygen species (27). Some of this may be related to which of its receptors are expressed and to changes in the types of NPY receptors with age (25, 27, 28). The finding that RPE produce both α-MSH and NPY and that the production of the two neuropeptides are required for RPE modulation of macrophage and microglial cell activity suggests that the eye is using a unique mechanism associated with the simultaneous stimulation of α-MSH and NPY receptors.

Recently, we have found that α-MSH and NPY cotreatment of macrophages does not suppress macrophage phagocytosis of opsonized materials but does suppress phagolysosome activation or formation (22). The phagocytic pathway in macrophages proceeds through identifiable steps of phagosome maturation with Rab5 replaced with Rab7 and then by LAMP1 as the phagosome fuses with the lysosome to make the phagolysosome (29). The vesicle’s proton pumps drop the pH within the phagolysosome to promote efficient proteolysis. Some of the peptides of the phagolysosome are shuttled to the MHC class II (MHC II) compartment and loaded onto MHC II molecules for presentation (30). Therefore, we assayed α-MSH/NPY–cotreated macrophages phagocytizing opsonized magnetic beads to determine if the suppression of phagolysosome activity is because of inhibition of phagosome maturation or phagolysosome formation. In addition, we assayed for changes in MHC II expression and Ag-presenting ability. Because we find that macrophages cotreated with α-MSH and NPY are suppressed in phagolysosome activation and in stimulating CD4^+^ T cell proliferation, suggesting that there is within the ocular microenvironment a mechanism to suppress conventional pathways of Ag processing. This would be another mechanism of ocular immune privilege to prevent effector CD4^+^ T cells clonally expanded in the periphery from seeing their presented Ags within the ocular microenvironment.

## MATERIALS AND METHODS

### Materials: cells, neuropeptides, and Abs

The macrophages in the experiments were the RAW 264.7 cell line (TIB-71) from the American Type Culture Collection (Manassas, VA.) and resting peritoneal macrophages from naive C57BL/6 mice. The macrophage cell line was cultured as instructed by the American Type Culture Collection, and cells were passaged three times after purchase, aliquoted, and stored at 2150°C. The macrophage cell line was passed three times before use after recovery from storage and kept in culture for no more than 3 mo and passed twice per week. Resting peritoneal macrophages were collected by peritoneal lavage of naive mice with 5 ml of 0.01M PBS (Lonza). The cells were centrifuged at 400g for 5 min, mixed with 1 ml of red cell lysing buffer (Sigma-Aldrich), iced for 5 min, and centrifuged at 400 × g for 5 min. The cells were resuspended in serum-free media (SFM) and used in the CD4 T cell stimulation assay. All animal studies conducted in this manuscript have been reviewed and approved by the Boston University Institutional Animal Care and Use Committee. The neuropeptides α-MSH (catalog number 4008476) and NPY (catalog number 4031105) were purchased from Bachem Americas (Torrance, CA). These neuropeptides were sorted in single-use aliquots at 1 mg/ml and stored at 280°C until used. The magnetic beads were tosylacti-vated M-280 Dynabeads from Thermo Fisher Scientific (Grand Island, NY) and the OVA fraction, V from Sigma-Aldrich (St. Louis, MO). The magnet was a microcentrifuge DYNAL magnetic holder (Thermo Fisher Scientific). The Ab used to opsonize the OVA-coated beads was a mouse monoclonal anti-OVA IgG_1_(2D11) from Santa Cruz Biotechnologies The Abs used for the immunoblotting were a mouse anti-Rab5(D-11), rabbit anti-Rab7(H-50), rat anti-mouse MHC II(IBL-5/22), and rat anti-LAMP1(1D4B) from Santa Cruz Biotechnology (Dallas, TX), and the same LAMP1 Ab was used for immunostaining the macrophages. A polyclonal rabbit anti-OVA from AbD Serotec was used for immunoblotting. Secondary Abs were donkey anti-mouse, anti-rat, and anti-rabbit conjugated to DayLight 488 or 649, and isotype controls were from Rockland (Limerick, PA). For flow cytometry, FITC-conjugated anti-mouse Ia^d^ from BD Biosciences (San Jose, CA) was used.

### Magnetic bead preparation

The M-280 beads (30 mg or 2 × 10^9^ beads) were added to 1 ml of borate buffer solution in a microcentrifuge tube and vortexed for 30 s. The tube was placed in a magnetic tube holder for 1 min, and the supernatant was removed. The tube was then removed from the magnet and resuspended in 1 ml of borate buffer solution. The tube was placed on the magnet, supernatant removed, and the beads resuspended in 300 µl of OVA (600 µg) in 150 mL of borate buffer solution. The mixture was incubated on a roller at room temperature for 24 h. Following incubation, the tube was placed on the magnet for 2 min, supernatant removed, and the beads were resuspended in 1 ml of PBS with 0.1% BSA and incubated at 37°C on a roller for 2 h. The tube was placed on the magnet, supernatant removed, and 1 ml of PBS was added again, the mixture was vortexed for 5–10 s, replaced on the magnet, and supernatant removed. This step was repeated, and then the beads were resuspended in 1 ml of PBS for storage at 4°C. The volume required for the experiment for 50 beads per cell was placed into a new microcentrifuge tube. This was placed on a magnet for 2 min, and the supernatant was removed. The beads were resuspended in PBS with 2 µl of 0.25 µg of mouse anti-OVA IgG_1_ per 25 µl of beads and incubated on a roller for 2 h. The tube was placed in the magnet, the supernatant removed, and the now-opsonized OVA magnetic beads were resuspended in SFM, ready to be added to the culture wells.

### Treatment, phagocytosis, and magnetic bead–containing vesicle isolation

The proteins of the phagocytic pathway vesicles were assayed by adaptation of published magnetic isolation methods (31–33). Macrophages (5 × 10^5^) were added into the wells of a 24-well tissue culture plate and incubated for 1 h. The cells were treated with α-MSH and NPY (1 ng/ml each) for 30 min, and into the wells was placed 2.5 × 10^7^ opsonized OVA magnetic beads. The cultures were incubated for 24 h, and to preserve intracellular vesicles, a 250 mM sucrose, 3 mM Imidazole, pH = 7.4 lysing buffer with protease inhibitors was used to lyse the cells (31). The magnetic bead–containing vesicles in the lysate were isolated using a magnet microfuge tube holder, washed twice, and resuspended with 0.1M PBS.

### Immunoblotting

The isolated vesicles were run on a 4–12% gradient NuPage gel, and the electrophoresed proteins were transferred to a non-fluorescent nylon filter. The filter paper was probed with Abs specific for OVA, Rab5, Rab7, Lamp1, and MHC II (H-2d). To quantify OVA degradation (Supplemental Fig. 1), the ratio of the intensity of whole OVA to the intensity of the OVA fragments was calculated. To measure changes in Rab7, Lamp1, and MHC II, the intensity of their bands was measured against the intensity of the Rab5 band.

### Cell staining

Immunocytochemistry was carried out on the macrophages to determine macrophage LAMP1 expression following treatment with α-MSH and NPY. The macrophages were cultured as described previously. Round coverslips were autoclaved and placed into a 24-well culture plate. A total of 2 × 10^4^ cells was placed on the coverslips (20 µl from a working concentration of 1 × 10^5^/ml), incubated for 2 h at 37°C with 5% CO_2_ atmosphere, and then 500 µl of SFM was added to each well. The macrophage cultures were divided into control (n = 3) and a-MSH/NPY (n = 3) groups. Opsonized pHrodo Red BioParticles were used to determine maturation of the phagolysosome (22). The *Staphylococcus* aureus pHrodo bioparticles (28 µl) were mixed with 28 µl of S. *aureus* bioparticle–opsonizing reagent, vortexed, and incubated on a roller at 37°C for 1 h. The mixture was then centrifuged at 1200 RPM for 15 min and washed in 200 µl of PBS twice, and the bioparticles were finally resuspended in 28 µl of SFM. The control wells received 10 µl of SFM and 2 µl of pHrodo bioparticles, and the α-MSH/NPY group received 5 µl of α-MSH (1 ng/ml), 5 µl of NPY (1 ng/ml), and 2 µl of pHrodo bioparticles. The negative control group received 12 µl of SFM with no neuropeptides and no pHrodo bioparticles. The cells were incubated for 24 h at 37°C, 5% CO_2_, fixed to the coverslips with 500 µl of 4% paraformaldehyde for 15 min and then given three 5-min washes in PBS on a rocker. Normal goat serum (5% in 1% Triton X-100/PBS) was added to each well to block nonspecific binding and incubated at room temperature for 30 min. A total of 300 µl of anti-LAMP1 IgG conjugated to Alexa Fluor488 diluted in 1% Triton X-100/PBS was added to each well for 24 h on a rocker at room temperature. The negative control received 1:50 rat IgG–Alexa Fluor488 in 1% Triton X-100/PBS. The cells were washed twice with PBS for 10 min on a rocker. The coverslips were removed from the wells and mounted on glass slides using 25 µl of DAPI/PVA-DABCO mounting media. The slides were left to be hardened and were imaged using an epifluorescent microscope. For both LAMP1 and pHrodo Red BioParticles, the corrected mean fluorescence was determined for each group (control *n* = 3, µ-MSH/NPY n = 3) using five fields of view per sample. Five readings of both background and fluorescence were taken per field of view to determine an average. This gave a total number of 15 mean corrected values per group for analysis.

### Flow cytometry

The macrophages were treated with a-MSH and NPY and fed opsonized nonfluorescent bioparticles as described above for 24 h, collected, and fixed in 5% paraformaldehyde. The macrophages were then resuspended in PBS with Fc block and incubated for 1 h at room temperature. The cells were washed and resuspended in PBS with monoclonal anti-mouse Ia^d^-FITC–conjugated Ab. The cells were then passed through a BD FACScan flow cytometer, and histograms were generated. From the histograms with background gated out (untreated macrophages fed opsonized bioparticles and stained treated with monoclonal isotype–FITC conjugate), the percentage of Ia^d^-positive cells was measured.

### CD4 T cell stimulation assay

This assay was optimized for maximum detection of Ag-stimulated T cell proliferation using the Cell Counting Kit-8 reagent (Dojindo Molecular Technologies, Rockville, MD) over background and cultures with no Ag. The collected peritoneal macrophages were plated onto a flat-bottom 96-well tissue culture plate (Corning, Corning, NY) at 7 × 10^5^ cells per well. The macrophages were treated with α-MSH and NPY at 1 ng/ml and incubated for 30 min at 37°C and 5% CO_2_. A total of 12.5 µg of opsonized OVA was added into the wells. The OVA was opsonized by mixing 200 µg of OVA with 0.25 µg of anti-OVA mouse IgG1 (Santa Cruz Biotechnology) incubated for 10 min room temperature. The Ag specificity control was that same amount of rat albumin (Sigma-Aldrich) mouse IgG1 (Santa Cruz) and anti-rat albumin instead of OVA and anti-OVA. The cultures were incubated 24 h at 37°C and 5% CO_2_. The APC cultures were washed twice with SFM, and 8×10^5^ OVA-primed T cells were added into the culture. The OVA-primed T cells were from draining popliteal lymph node of mice immunized with OVA (200 µg/mouse) in CFA (Difco Labs, Detroit, MI). The T cells were isolated using a CD4 T cell isolation column from R&D Systems (Minneapolis, MN). The T cell cultures were incubated for 72 h at 37°C and 5% CO_2_. Into each well was added Cell Counting Kit-8 reagent, and the cultures were further incubated for 3 h. The absorbance was measured of each well at 450 nm. The levels of absorbance were calculated relative to the level of absorbance of the positive-culture well (OVA-presenting APC with OVA-primed T cells).

### Statistical analysis

The data from at least three independent experiments were tabulated, analyzed, and graphed as mean ± SEM by GraphPad Prism software. Control results were compared with the α-MSH/NPY cotreatment groups using a one-way ANOVA with a two-tailed Dunnett assay for multiple comparisons to determine statistical significance. Differences were considered significant when *p* ≤ 0.05.

## RESULTS

### The effects of a-MSH/NPY cotreatment on phagosome maturation

**FIGURE 1.**
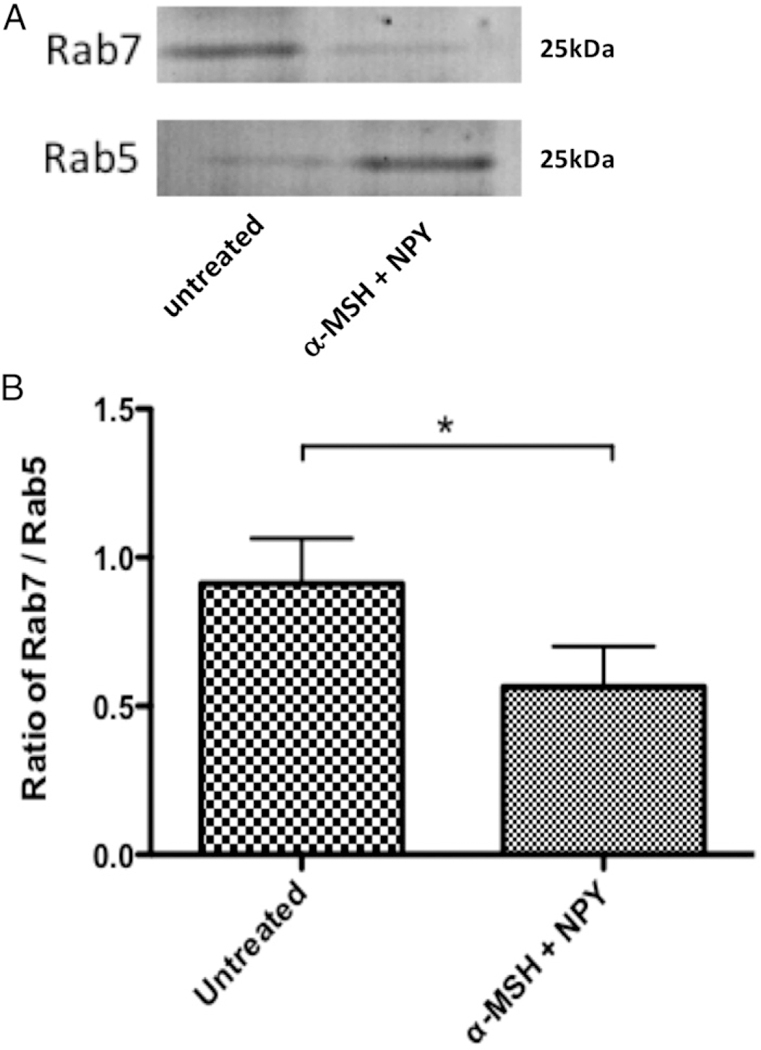
The effects of neuropeptide treatment on phagosome maturation. The isolated magnetic bead–containing vesicles were immunoblotted for Rab5 and Rab7. The ratio of Rab7 to Rab5 was calculated from the intensity of the bands. Presented are (**A**) a representative immunoblot and (**B**) the mean ± SEM of the relative ratio of Rab7/Rab5 from three independent experiments. *There is a highly significant (*p* = 0.008) suppression in the expression of Rab7 to Rab5 in the macrophages cotreated with α-MSH and NPY. This demonstrates suppression in the maturation of the phagosomes.

Our previous work demonstrated that α-MSH/NPY cotreatment of macrophages does not suppress uptake of opsonized material; however, detection of activated phagolysosomes was suppressed (22). One possibility is that the neuropeptide treatment affects the intracellular maturation pathway of the phagosomes. To detect suppression in the maturation of the phagosomes, the RAW264.7 macrophages were treated with both α-MSH and NPY and fed opsonized OVA magnetic beads. After 24 h incubation, the macrophages were lysed and the magnetic bead–containing intracellular vesicles (the phagosomes and phagolysosomes) were isolated using a magnet. The isolated intracellular vesicles were analyzed by immunoblotting for Rab5 and Rab7 (Fig. 1A). There was significant suppression in the ratio of Rab7 versus Rab5 (Fig. 1B). This indicates that the neuropeptide treatment suppressed the phagosome maturation pathway at the transition from early Rab5-containing phagosomes to the intermediate/late phagosome where Rab5 is replaced with Rab7.

### The effects of α-MSH/NPY cotreatment on phagolysosome formation

**FIGURE 2.**
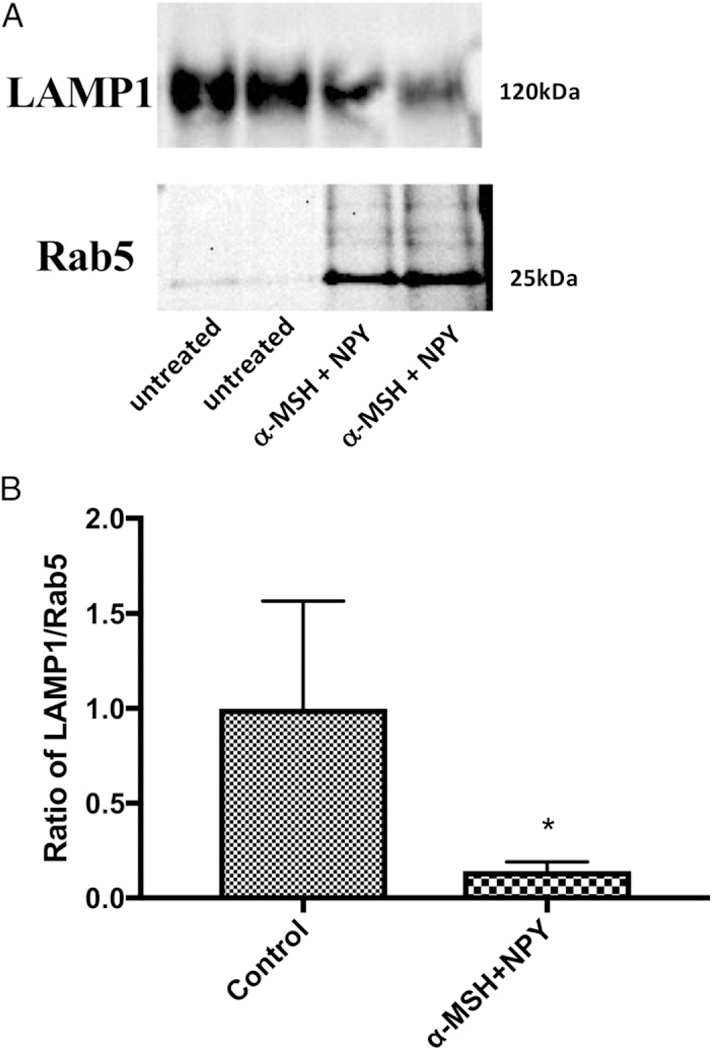
The effects of neuropeptide treatment on phagolysosome formation. The isolated magnetic bead–containing vesicles were immunoblotted for Rab5 and LAMP1. The ratio of LAMP1 to Rab5 was calculated from the intensity of the bands. Presented are (**A**) a representative immunoblot and (**B**) the mean 6 SEM of the relative ratio of LAMP1/Rab5 from four independent experiments. *There is a significant (*p* = 0.03) suppression in the expression of LAMP1 to Rab5 in the macrophages cotreated with α-MSH and NPY. This demonstrates suppression in the formation of phagolysosomes.

Another possibility for not seeing activated phagolysosomes in the neuropeptide-treated macrophages is that phagolysosome formation and activation was suppressed. The isolated intracellular vesicles were analyzed by immunoblotting for Rab5 and LAMP1 (Fig. 2A). There was significant suppression in the ratio of LAMP1 to Rab5 (Fig. 2B), indicating that α-MSH/NPY cotreatment inhibits phagolysosome formation. Moreover, the presence of Rab5 in the isolated phagosomes of the α-MSH/NPY-cotreated macrophages in comparison with the diminished presence of Rab5 in the phagosomes of the untreated macrophages further demonstrates that the phagosomes are being held in the early stages of phagosome maturation within the neuropeptide-treated macrophages.

**FIGURE 3.**
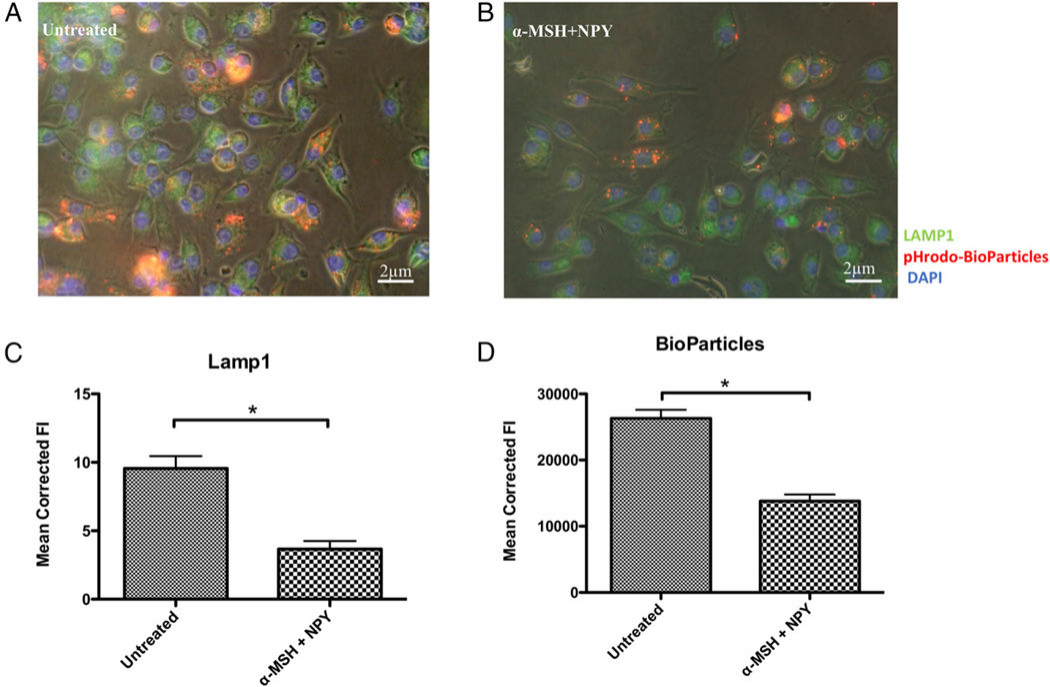
The effects of neuropeptide treatment on LAMP1 expression. The macrophages were fed opsonized pHrodo–*S*. *aureus* bioparticles, fixed, and stained for LAMP1 and DAPI. Representative images of (**A**) untreated and (**B**) α-MSH/NPY–cotreated macrophages. (**C** and **D**) The bar graphs are the mean fluorescent intensities ± SEM of 15 independent experiments. This demonstrates a suppression in LAMP1 protein production in the treated macrophages. *There was a highly significant (p ± 0.001) suppression of LAMP1 expression (C) in the α-MSH/NPY–cotreated macrophages, with a corresponding suppression in pHrodo Red BioParticle intensity (D).

The neuropeptide-treated macrophages were stained for LAMP1 after they phagocytosed opsonized pHrodo Red BioParticles (Fig. 3). There was an observable (Fig. 3A) and quantifiable (Fig. 3B) suppression in LAMP1 protein expression in the α-MSH/ NPY-cotreated macrophages. It corresponded with equally suppressed fluorescence of pHrodo Red BioParticles. Therefore, be-sides the potential for the neuropeptides to suppress the maturation of the phagosome, they also suppress the expression of LAMP1 that affects activation of lysosomal activity.

### The effects of α-MSH/NPY cotreatment on Ag processing

**FIGURE 4.**
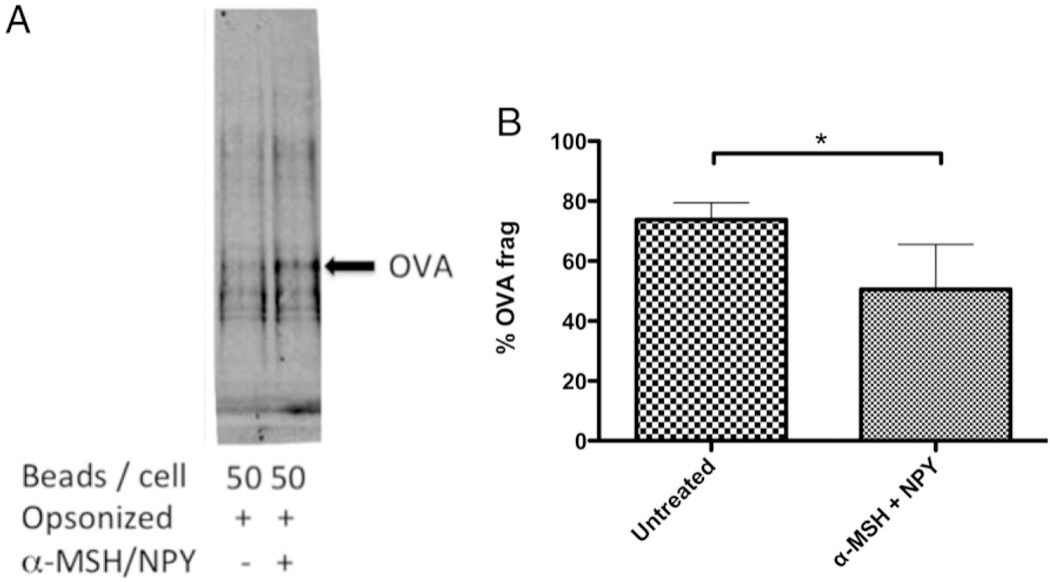
Effects of α -MSH/NPY cotreatment on OVA fragmentation within macrophages. (**A**) The isolated magnetic bead–containing vesicles were immunoblotted for OVA. (**B**) The percentage of OVA fragmented was calculated by the ratio of the intensities of OVA fragments over the sum of the fragments plus whole OVA band intensities. Significant suppression of OVA frag-mentation was detected in macrophages cotreated with α-MSH and NPY. Presented are the mean ± SEM of the percent OVA fragments (% OVA frag) of three independent experiments, with *p ≤ 0.01.

Because the acidification of phagolysosomes and the maturation pathway of phagosomes is suppressed in α-MSH/NPY– cotreated macrophages phagocytizing bioparticles, the expected degradation of phagocytized proteins would also be suppressed. The macrophages were treated with α-MSH and NPY and then fed opsonized OVA magnetic beads. After 24 h incubation, the cells were lysed, and the intracellular vesicles were isolated using a magnet. The isolated intercellular vesicles were analyzed by immunoblotting using a rabbit anti-OVA Ab. The immunoblotting demonstrated both intact and fragments of processed OVA (Fig. 4A). When the macrophages were cotreated with α-MSH and NPY, there was a significant reduction in the detection of OVA fragments (Fig. 4B). Therefore, the suppression of the activation of the phagolysosome by α -MSH/NPY cotreat-ment is associated with diminished processing of phagocytized Ag.

### The effects of α-MSH/NPY cotreatment on macrophage expression of MHC II

**FIGURE 5.**
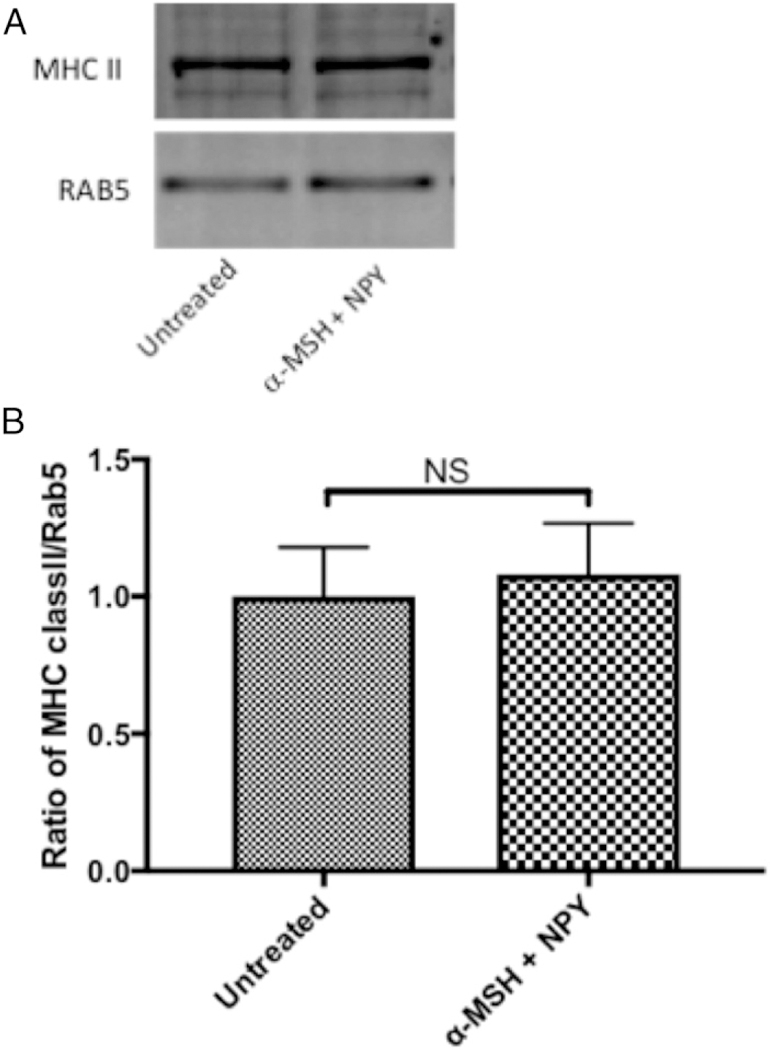
The effects of neuropeptide treatment on MHC II within magnetic bead–containing vesicles. The isolated magnetic bead–containing vesicles were immunoblotted for Rab5 and MHC II. The ratio of MHC II to Rab5 was calculated from the intensity of the bands. Presented are (**A**) representative immunoblot and (**B**) the mean ± SEM of the relative ratio of MHC II/Rab5 from three independent experiments. There was no significant suppression in the expression of MHC II to Rab5 in the macrophages cotreated with α-MSH and NPY. This demonstrates that MHC II is still associated with phagosomes.

**FIGURE 6.**
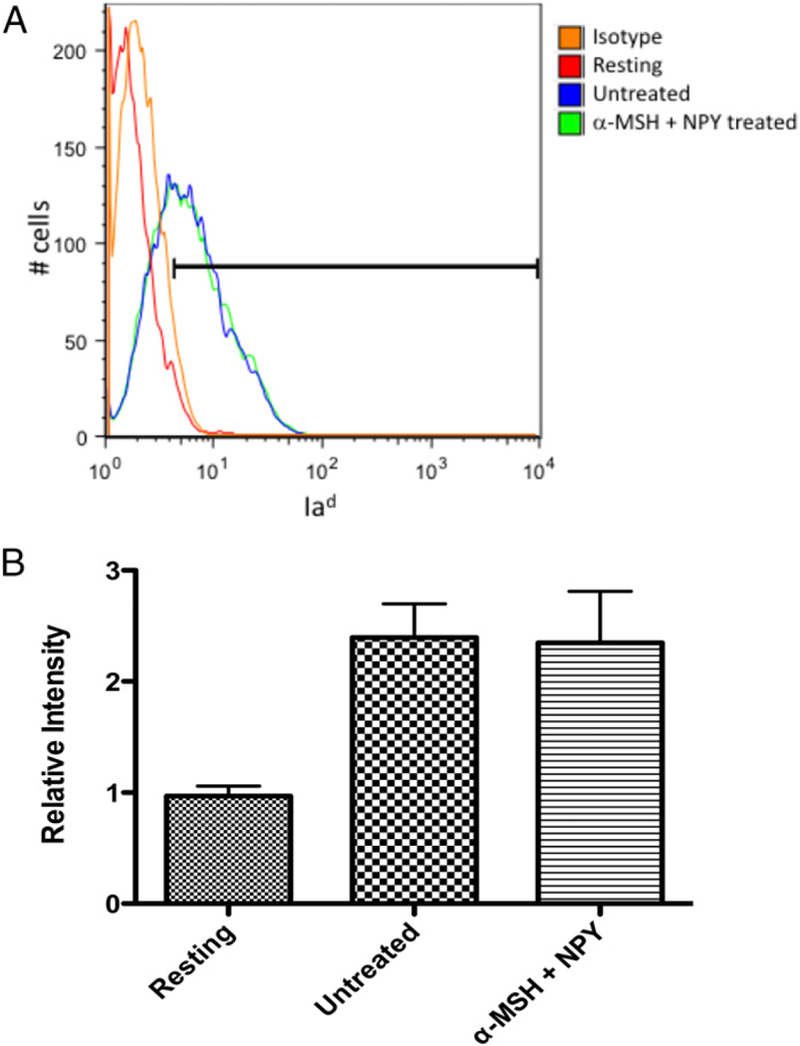
Effects of neuropeptide treatment on Ia^d^ expression on macrophages. The α-MSH/NPY–cotreated macrophages were fixed and stained for surface expression of Ia^d^ and analyzed by flow cytometry. (**A**) Repre-sentative histogram of three independent experiments with the gate set to exclude ˃.95% of isotype-stained cells. (**B**) The bar graph presents the relative intensity of Ia^d^ expression on untreated and α-MSH/NPY– cotreated macrophages given opsonized nonfluorescent bioparticles to phagocytize, and resting macrophages. No significant difference was seen between activated macrophages treated or untreated in Ia^d^ ex-pression. This demonstrates that MHC II expression is not altered in the treated macrophages.

The conventional pathway for processed phagocytized materials is presentation on MHC II molecules. Because there was a significant suppression in the phagocytic pathway and phagolyso-some activation by α -MSH/NPY cotreatment, there may be an effect on MHC II expression. The isolated intracellular vesicles were analyzed by immunoblotting for Rab5 and MHC II (Fig. 5A). There was no suppression in ratio of MHC II to Rab5 (Fig. 5B), indicating that α -MSH/NPY cotreatment does not prevent MHC molecules to be in the phagosomes. Flow cytometry was done and showed that there was no change of constitutive surface expression of MHC II on the neuropeptide-treated macrophages (Fig. 6). This suggests that although the processing of phagocytized Ag may be altered by 03b1-MSH/NPY cotreatment, the macrophages can still act as Ag-presenting cells.

### The effects of α -MSH/NPY cotreatment on APC stimulation of Ag-specific T cell proliferation

**FIGURE 7.**
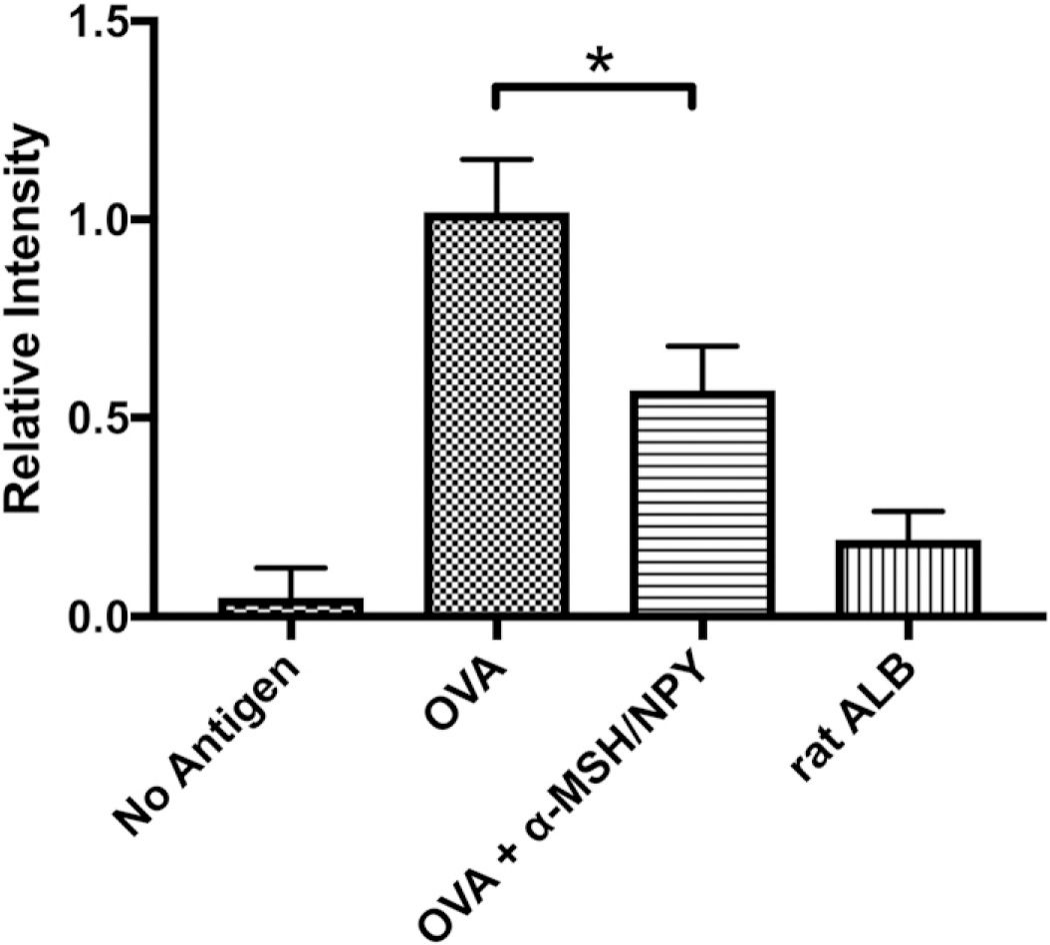
Effects of neuropeptide treatment on APC stimulation of Ag-specific T cells. The α-MSH/NPY–cotreated macrophages were fed opsonized OVA to process. OVA-specific T cells were added to the wells and assayed for proliferation. Presented are the relative levels of proliferation in comparison with the proliferation detected in the cultures of untreated OVA-presenting APC with OVA-specific T cells (OVA). Wells of no Ag contained only cultured APC with OVA-specific T cells. For Ag specificity, opsonized rat albumin was used instead of opsonized OVA (ratALB). The results presented are from nine independent cultures. This demonstrates that the treated APC are suppressed in their ability to stimulate Ag-specific T cell proliferation. The neuropeptide-treated APC were significantly suppressed (*p ≤ 0.001) in their ability to stimulate T cell proliferation.

Because we demonstrate that the neuropeptide-treated macro-phages have an altered phagocytic pathway but continue to express MHC II, we assayed the potential for the treated macrophages to be APC and to Ag stimulate CD4^+^ T cell proliferation. For these experiments, resting peritoneal macrophages were treated with α -MSH and NPY and fed opsonized OVA. After incubation, the cells were used as APC and mixed with OVA-primed CD4^+^ T cells, and proliferation was assayed (Fig. 7). There was significant suppression in the ability of the treated APC to stimulate T cell proliferation in comparison with untreated APC fed opsonized OVA. This demonstrates that α-MSH/NPY–treated APC are suppressed in their ability to Ag stimulate CD4 T cell proliferation.

## DISCUSSION

The mechanisms of ocular immune privilege are mediated in part by neuropeptides constitutively present and produced within the ocular microenvironment. Two of these neuropeptides, α-MSH and NPY, coexpressed within the healthy eye and produced by RPE, affect the functionality of macrophages (3). Together, these neuropeptides induce expression of suppressor cell characteristics in macrophages, and as presented in this manuscript, alter the phagocytic pathway to phagolysosome activation. The neuropeptide-treated macrophages were suppressed in phagosome maturation. In addition, there was suppression in LAMP1, which impairs phagolysosome activation.Although the presence ofMHCII within thephagosomesdid not change, therewas suppression in the processing of OVA and in the ability of the macrophages to act as APC in stimulating CD4 T cell proliferation. Ag processing would have to be through an alternative pathway of neutralpHproteolysis of proteins. This suggests that a potential mechanism of ocular immune privilege is through the effects of α-MSHandNPYonAPC to present peptides not recognized by effector T cells expanded in extraocular sites to Ags processed through conventional pathways.

The neuropeptide-treatedmacrophages retained expressionof MHC II, meaning that they retain Ag-presenting capabilities. It has been known that F4/80-positive macrophages when treated with aqueous humor mediate activation of regulatory T (Treg) cells (4, 16, 18, 34–36). Recently Lee et al. (37) demonstrated that macrophages treated with α-MSH convert autoreactive effector T cells into Treg cells. Moreover, it is these macrophages that expand in the spleen at the resolution of experimental autoimmune uveitis and are dependent on the expression of the α-MSH receptor MC5r. Therefore, the continued expression of MHC II was expected; however, because the phagosomes do not mature, it is likely that the source of MHC II in the phagosomes is not from the MHC II compartment but from recirculated MHC II taken up during phagocytosis.

Early phagosomes have the ability to degrade phagocytized proteins (38, 39). This would mean that there remains the possibility of new peptides being loaded on the recirculated MHC II. The Ag processing would be under neutral pH conditions, suggesting a different set of processed peptides than what would be expected if the peptides were processed in the pH4 phagolysosome. The implications of this possibility would be that through α-MSH/NPY, ocular immune privilege has a mechanism of diverting the processing of autoantigens away from T cell–recognizable peptides to unique unrecognizable peptides.

Individually the two neuropeptides have been demonstrated to regulate phagocytosis and maturation of phagosomes. It has been demonstrated that α-MSH and NPY each suppress macrophage uptake of unopsonized bioparticles but not opsonized FcR-mediated phagocytosis (22). Such facilitated phagocytosis is linked to activation of proinflammatory activity by macrophages; however, the α-MSH–only–treated macrophages are anti-inflammatory in activity, and the α-MSH/NPY–cotreated macro-phages express characteristics of myeloid suppressor cells (2, 3, 40). In contrast, the NPY-only–treated macrophages express M1 macrophage activity (3). The effects of NPY on phagocytosis is mixed as to whether it is suppressive or stimulating. Some of these differences may involve the pathogen being phagocytized and age of the mouse (21, 24, 26, 28). Compounding the under-standing of the activity of α-MSH and NPY on phagocytosis is that there are multiple receptors for the two neuropeptides. The melanocortin 1 and 3 receptors appear associated with α-MSH regulation of innate immune activity of macrophages, and Y1 and Y2 receptor subtype with NPY effects on phagocytosis (25, 41). Most of these studies looked at the uptake and not necessarily the phagosome maturation pathway. The results in this manuscript with our previous publication showing suppression of the phagolysosome activation (5, 6, 22) demonstrates that it is not the uptake but the maturation of the phagocytic pathway that is regulated by α-MSH and NPY.

The α-MSH/NPY cotreatment suppression of Rab7 recruitment and exchange on the phagosome would move the phagosome to a recycling pathway instead of moving onto late-state phagosomes that would fuse with the lysosome and then onto the MHC II compartment (29, 42). Suppressed LAMP1 expression means that the effects of α-MSH/NPY cotreatment is both suppression of phagosome maturation and inhibition of phagoly-sosome activation. This finding has implications on macrophages to mediate intracellular autophagy and possibly processing phagocytized materials for energy. More importantly, it affects the processing of phagocytized Ags into peptides for loading onto MHC II for presentation.

The ocular microenvironment expresses simultaneously both α-MSH and NPY produced by healthy RPE. The effects of these neuropeptides induce myeloid suppressor cell characteristics in macrophages, and retinal microglial cells express the same characteristics (3). These macrophages, when stimulated with endotoxin, produce anti-inflammatory cytokines, are poor in Agactivating effector T cells, and in a nonantigen–specific manner may induce apoptosis in the T cells (2, 24). In contrast, macro-phages influenced by the ocular microenvironment are highly efficient in activating Ag-specific Treg cells within the spleen of mice (34, 43). This is an example of the ocular microenviron-ment programming innate cells to regulate at the interface of innate and adaptive immunity. Therefore, within the healthy ocular microenvironment through the constitutive expression of both α-MSH and NPY, there would be an expectation of suppressed phagosome maturation that prevents conven-tional processing of autoantigens. This may be a subtle mechanism of Ag sequestration within the immune privileged ocular microenvironment.

## Supplementary Material

Fig. S1

## Abbreviations used in this article:

MHC II: MHC class II
α-MSH: α-melanocyte–stimulating hormone
NPY: neuropeptide Y
RPE: retinal pigment epithelial cell
SFM: serum-free media
Treg: regulatory T
