# Supplementary figures and images for: The Neuropeptides of Ocular Immune Privilege, α-MSH and NPY, Suppress Phagosome Maturation in Macrophages
Source: Immunohorizons. Author manuscript; Available in PMC 2019 Jan 4. (PMC6319950; doi:10.4049/immunohorizons.1800049)

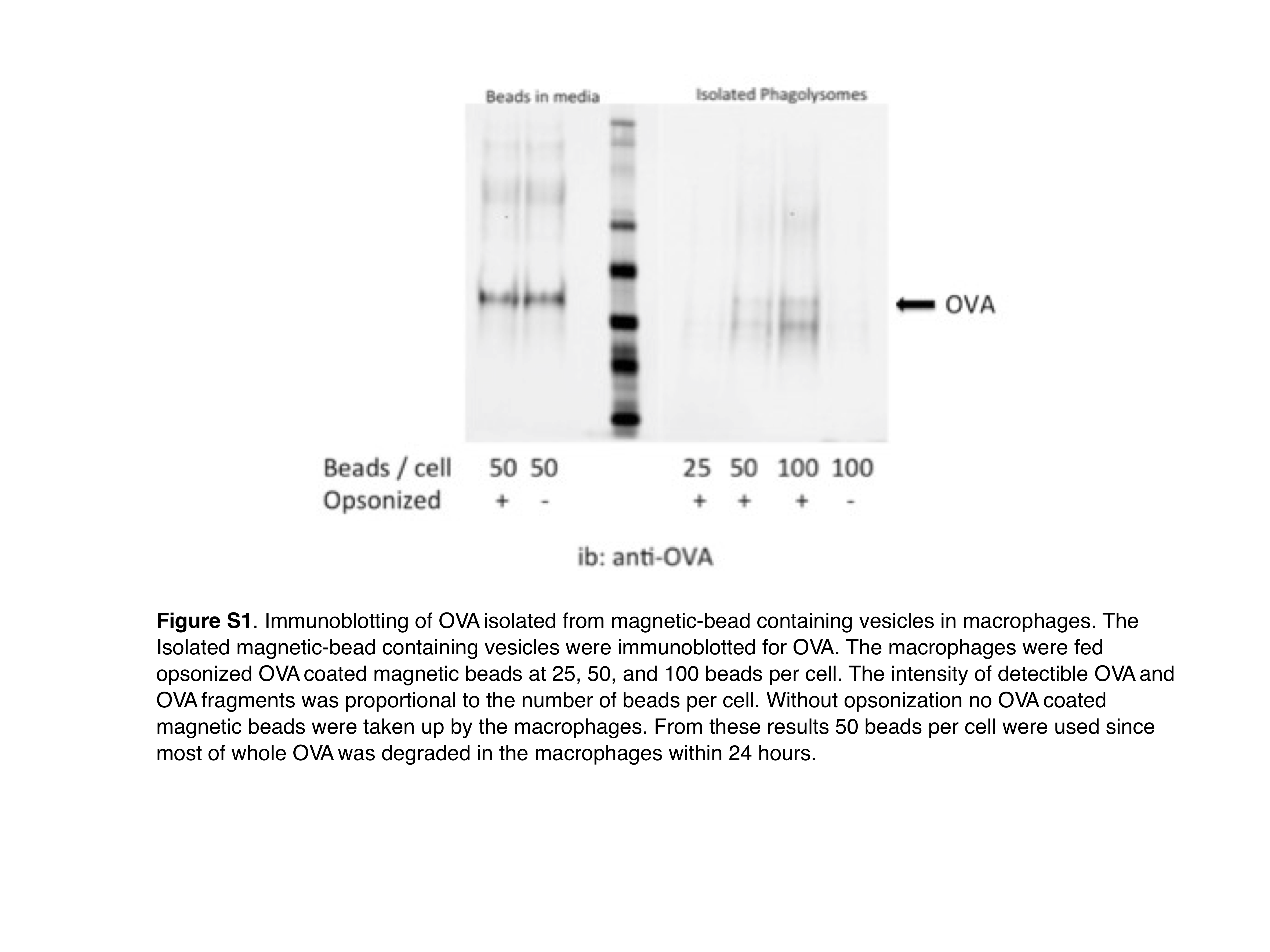

Supplement: Fig. S1 [file NIHMS998071-supplement-Fig__S1.tiff]
